# Effect of bariatric surgery on mitochondrial remodeling in human skeletal muscle: a narrative review

**DOI:** 10.3389/fendo.2024.1488715

**Published:** 2024-11-25

**Authors:** Xiaochuan Ge, Zhe Wang, Yafeng Song, Hua Meng

**Affiliations:** ^1^ Department of Exercise Physiology, Beijing Sport University, Beijing, China; ^2^ Key Laboratory of Sports and Physical Fitness of the Ministry of Education, Beijing Sport University, Beijing, China; ^3^ Department of General Surgery & Obesity and Metabolic Disease Center, China-Japan Friendship Hospital, Beijing, China

**Keywords:** obesity, type 2 diabetes, weight-loss surgery, skeletal muscle mitochondria, metabolism

## Abstract

In the context of obesity epidemic as a major global public health challenge, bariatric surgery stands out for its significant and long-lasting effectiveness in addressing severe obesity and its associated comorbidities. Skeletal muscle mitochondrial function, which is crucial for maintaining metabolic health, tends to deteriorate with obesity. This review summarized current evidence on the effects of bariatric surgery on skeletal muscle mitochondrial function, with a focus on mitochondrial content, mitochondrial dynamics, mitochondrial respiration and mitochondrial markers in glucolipid metabolism. In conclusion, bariatric surgery impacts skeletal muscle through pathways related to mitochondrial function and induces mitochondrial remodeling in skeletal muscle in various aspects. Future studies should focus on standardized methodologies, larger sample sizes, and better control of confounding factors to further clarify the role of mitochondrial remodeling in the therapeutic benefits of bariatric surgery.

## 1 Introduction

Obesity prevalence has increased worldwide in the past 50 years (1), and this trend is expected to continue in the near future. According to World Obesity Atlas 2023, in 2020, 38% of the global population were classified as overweight or obese, and this rate was predicted to reach 51% by 2035, affecting over 4 billion individuals (2). As obesity significantly increases the risk of multiple chronic diseases and mortality (3, 4), the alarming rise in this epidemic poses a major public health challenge and substantial economic burden worldwide. It is estimated that the economic impact of overweight and obesity will rise from US$1.96 trillion in 2020 to US$4.32 trillion by 2035, resulting in a reduction in global GDP from 2.4% to 2.9% (2). All these factors highlight the urgent need for effective strategies to address this global health issue.

In combating obesity, lifestyle intervention, pharmacotherapy, and bariatric surgery are the three major therapeutic options. Among these, bariatric surgery stands out for its significant and sustainable effects in treating severe obesity and its related comorbidities. While lifestyle intervention and medications provide some benefits, bariatric surgery often leads to the most substantial and lasting weight loss outcomes (5, 6). There are four common bariatric procedures performed worldwide: laparoscopic sleeve gastrectomy (SG), laparoscopic Roux-en-Y gastric bypass (RYGB), laparoscopic adjustable gastric banding (LAGB) and biliopancreatic diversion (BPD) (7). RYGB and SG are the two most widely used surgical procedures, while LAGB and BPD are less frequently employed (7). In terms of weight loss effectiveness and the degree of type 2 diabetes (T2D) remission, BPD yields the best results (although with higher complication rates and mortality), followed by RYGB, then LSG, with LAGB showing the least effectiveness (8, 9).

As one of the most metabolically active tissues, skeletal muscle constitutes a substantial percentage of total body mass (10) and plays an important role in metabolic health. First, skeletal muscle is a major determinant of whole-body energy expenditure. Through aerobic metabolism, it consumes 20-30% of the body’s oxygen uptake at rest, which can rise to 90% during exercise (11). In addition, skeletal muscle is the largest insulin-sensitive tissue in the body and the primary site for insulin-stimulated glucose utilization, accounting for ~80% postprandial glucose uptake (12). As the powerhouse of the skeletal muscle, mitochondria are the main sites of oxygen consumption, where glucose and lipids are converted into ATP through aerobic metabolism. Therefore, skeletal muscular mitochondrial function is closely linked to the metabolic state of both skeletal muscle and the whole body. On the other hand, reduced mitochondrial function within skeletal muscle is often associated with metabolic disorders, such as obesity, insulin resistance and T2D. Evidence has shown that mitochondrial function is impaired in the skeletal muscle of obese individuals (13, 14), which may contribute to the development of skeletal muscle insulin resistance (15, 16). The impaired mitochondrial function may manifest as reduced mitochondrial content and enzyme activity, alterations in mitochondrial network structure, and changes in respiratory capacity (17).

Although bariatric surgery results in a substantial decrease in skeletal muscle mass as part of the weight loss process (18), it simultaneously improves metabolic disorders in skeletal muscle and overall body, as evidenced by a significant enhancement in insulin sensitivity (19). Given the close relationship between the skeletal muscular mitochondria and energy substrate metabolism, this review focused on the impact of bariatric surgery on mitochondrial remodeling in skeletal muscle. Specifically, we examined changes in mitochondrial content, mitochondrial dynamics, mitochondrial respiration and several other key mitochondrial markers. The aim of this article is to provide insights into the mitochondrial-related mechanisms through which bariatric surgery improves metabolic health in obese individuals, and to highlight areas requiring further investigation to fully elucidate the role of mitochondrial remodeling in the therapeutic benefits of bariatric surgery.

## 2 Literature search and assessment of study quality

**Figure 1:**
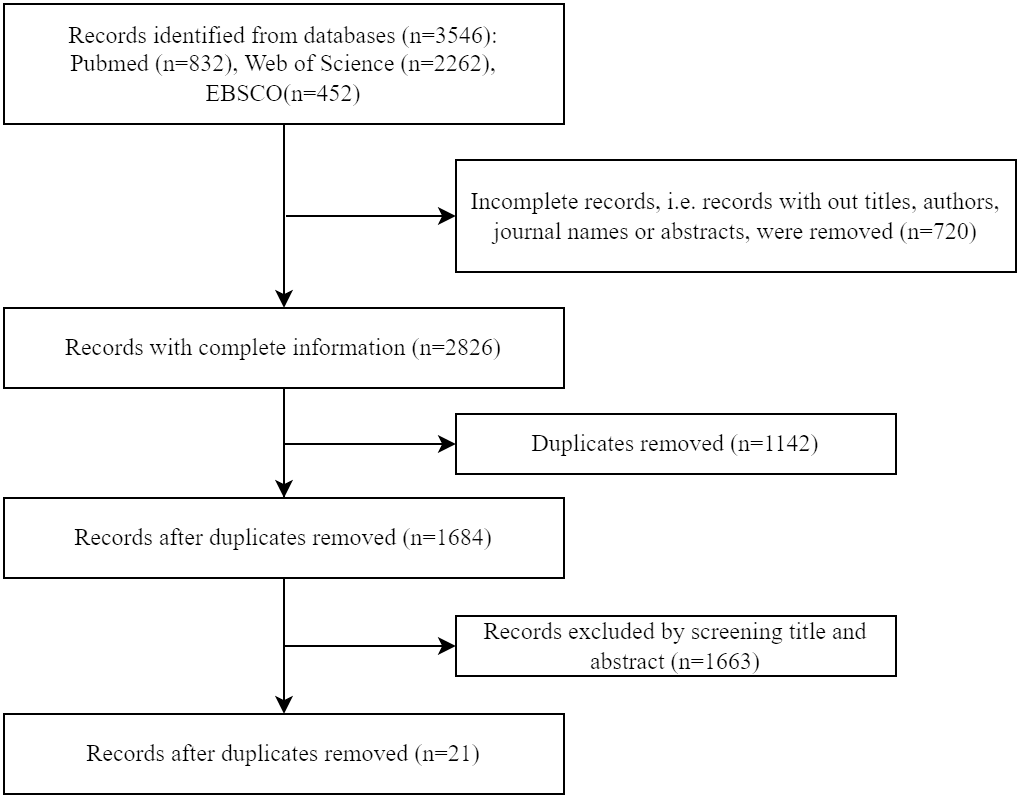
Flow diagram for literature search and study selection.

A literature search was conducted using Pubmed, Web of Science and EBSCO databases on 22 March 2024. The terms combining the concepts of “bariatric surgery” and “muscle” were searched in their synonyms to ensure a broad scope (see detailed search strategies in **Supplementary Table 1**). All articles were screened based on their title and abstract, and studies examining skeletal muscle mitochondria in humans following bariatric surgery were included and systematically reviewed in a descriptive manner. Animal studies, conference abstracts, books, comments, letters, case reports, editorials, and non-English articles were excluded. A total of 3,546 articles were identified, and 21 relevant studies were selected for the next step. (**Figure 1**)

The revised JBI (Joanna Briggs Institute) critical appraisal tool for quasi-experimental studies (20) was applied to evaluate the quality of the included studies. Our primary intervention of interest is bariatric surgery, and the outcome measures focus on changes in mitochondrial morphology and functional parameters, or related molecules with in mitochondria. Based on this, two reviewers independently assessed the study quality, and any disagreements were resolved through discussion. As presented in **Table 1**, most studies received more than five “yes” during the assessment and were included in this review. The only study that received fewer than five “yes” had some erroneous results excluded, while the remaining valid part were still included in the review, which will be clearly indicated later.

**Table 1:** Quality assessment using JBI (Joanna Briggs Institute) critical appraisal tool for quasi-experimental studies.

Study	Q1	Q2	Q3	Q4	Q5	Q6	Q7	Q8	Q9	Total
M. D. Barberio et al. (21)	Y	N	N	U	Y	Y	Y	N/A	Y	5/9
B. A. Kugler et al. (46)	Y	N	Y	U	Y	Y	Y	N/A	Y	6/9
S. Gancheva et al. (24)	Y	N	N	U	Y	Y	Y	N/A	U	4/9
M. T. Lund et al. (28)	Y	N	N	U	Y	Y	Y	N/A	Y	5/9
M. D. Kristensen et al. (14)	Y	N	N	U	Y	Y	Y	N/A	Y	5/9
C.S. Mehnert et al. (27)	Y	N	U	U	Y	Y	Y	N/A	Y	5/9
J. M. Hinkley (69)	Y	N	Y	U	Y	Y	Y	N/A	Y	6/9
L. E. Campbel et al. (23)	Y	N	N	U	Y	Y	Y	N/A	Y	5/9
M. Fernström et al. (60)	Y	N	Y	Y	Y	Y	Y	N/A	Y	7/9
E. B. M. Nascimento (70)	Y	N	Y	U	Y	Y	Y	N/A	Y	6/9
R. Barres et al. (22)	Y	N	Y	U	Y	Y	Y	N/A	Y	6/9
G. H. Vijgen et al. (62)	Y	N	Y	U	Y	Y	Y	N/A	Y	6/9
S. Nijhawan et al. (61)	Y	N	N	U	Y	Y	Y	N/A	Y	5/9
M. I. Hernández-Alvarez et al. (45)	Y	N	N	U	Y	Y	Y	N/A	Y	5/9
G. Gastaldi et al. (47)	Y	N	N	U	Y	Y	Y	N/A	Y	5/9
G. Mingrone et al. (43)	Y	N	Y	U	Y	Y	Y	N/A	Y	6/9
D. Bach et al. (44)	Y	N	Y	U	Y	Y	Y	N/A	Y	6/9
G. Mingrone et al. (71)	Y	N	Y	U	Y	Y	Y	N/A	Y	6/9
R. Fabris (68)	Y	N	Y	U	Y	Y	Y	N/A	Y	6/9
G. Mingrone et al. (56)	Y	N	Y	U	Y	Y	Y	N/A	Y	6/9
G. Rosa (67)	Y	N	Y	U	Y	Y	Y	N/A	Y	6/9

Q1–Q9: Questions based on JBI critical appraisal tool for quasi-experimental studies, see reference (20); Y, yes; N, no; U, unclear; N/A, not applicable; Total, number of “yes” of all 9 question.

## 3 Results

### 3.1 Mitochondrial remodeling: insights from transcriptomic and proteomic analyses

Bariatric surgery has been a focus of intense research as an effective method to improve metabolic health, including skeletal muscle health. Four studies (21–24) investigated skeletal muscle transcriptome and/or proteome to provide a comprehensive and unbiased understanding of muscle adaptation following surgery. However, one study (24, 25) was excluded due to errors in transcriptomic analysis.

As shown in **Table 2**, although the number of differentially expressed genes varied considerably between obese and healthy lean groups, as well as between pre- and post-surgery conditions, all studies consistently reported the enrichment of mitochondria-related processes or functions. Furthermore, these studies investigated the transcriptomic and/or proteomic responses at 3 months (23), 6 months (22), and 1 year (21) after bariatric surgery, respectively. The variation in results across studies may indicate a gradual restoration of mitochondrial function over time. Campbell (23) found that oxidative phosphorylation and the citrate cycle were downregulated in obese patients prior to surgery, with these processes remaining unchanged 3 months post-surgery. However, improved mitochondrial function was observed at 6 months in Barres’ study (22) and at 1 year in Barberio’s study (21) following RYGB. Nonetheless, it is important to note that the small sample sizes and diverse approaches employed in transcriptomic/proteomic analysis may have contributed to the observed differences in results. Therefore, further investigation is warranted to reanalyze and synthesize the omics data from these studies using standardized bioinformatic methods before drawing definitive conclusions.

**Table 2:** Transcriptomic and proteomic characteristics of skeletal muscle following bariatric surgery.

Author (Year) (Reference)	Subjects information		Type of surgery & Time points	Site of skeletal muscle & Sample type	Main Finding	
	OG	LG				
M. D. Barberio (2021) (21)	n=7 (F; Non-T2D) n=5 (F; T2D)	N/A	RYGB **PRE** **POST**: 1y	Vastus lateralis Ex-vivo tissue	**Differentially expressed genes (probes)** - OG[PRE(T2D VS Non-T2D)]: 97 - OG[POST(T2D VS Non-T2D)]: 3 - OG[T2D(POST VS PRE)]: 53 - OG[Non-T2D(POST VS PRE)]: 11	Bioinformatics of transcriptome* OG[PRE(T2D VS Non-T2D)]: - Mitochondrial Dysfunction - Oxidative Phosphorylation OG[T2D(POST VS PRE)]: - Oxidative Phosphorylation - Mitochondrial Dysfunction
L. E. Campbell (2016) (23)	n=7 (F; T2D & Non-T2D)	n=4 (F)	RYGB **PRE** **POST**: 3m	Vastus lateralis Ex-vivo tissue	**Differentially expressed proteins** - OG(PRE) VS LG: 260↓, 135↑ - OG[POST VS PRE)]: 52↓, 228↑ - [OG(PRE) VS LG] & [OG(POST VS PRE)]: 49 **Differentially expressed genes (probes)** - OG(POST VS PRE): 3047↑, 2643↓	Bioinformatics of preteome** OG(PRE) VS LG: - hsa00190: Oxidative phosphorylation ↓ - hsa00020: Citrate cycle (TCA cycle) ↓ Bioinformatics of transcriptome** OG(POST VS PRE): - hsa00190: Oxidative phosphorylation ↓ - hsa00020: Citrate cycle (TCA cycle) ↓
R. Barres (2013) (22)	n=5 (F; Non-T2D)	n=6 (F)	RYGB **PRE** **POST**: 6m	Vastus lateralis Ex-vivo tissue	**Differentially expressed genes** - OG(POST VS PRE): 896 - OG(PRE) VS LG: 274 - OG(POST) VS LG: 38	Bioinformatics of transcriptome*** OG(POST VS PRE): - Lipid metabolic process (GO:0006629) - Mitochondrion (GO:0005739)

OG, Obese Group; LG, Lean Group; F, Female; M, Male; T2D, Type 2 Diabetes; Non-T2D, without Type 2 Diabetes; N/A, not applicable; RYGB, Roux-en-Y gastric bypass; PRE, Pre-surgery; POST, Post-surgery; y, year or years; m, month or months; ↑, upregulation; ↓, downregulation.

* Ingenuity Pathway Analysis using Canonical Pathway tool.

** Gene Ontology (GO) Enrichment Analysis.

*** KEGG pathway analysis.

### 3.2 Mitochondrial content remodeling

Mitochondrial content typically serves as an indicator of mitochondrial oxidative capacity. While transmission electron microscopy (TEM) is considered the gold standard for assessing mitochondrial content, alternative methods utilizing mitochondrial biomarkers are often employed for convenience. Larsen et al. (26) demonstrated that cardiolipin and citrate synthase activity (CSA) ranked as the top two strongest markers associated with mitochondrial content. The activity or content of several respiratory chain components, which are also closely linked to mitochondrial content (26), will be discussed in other sections of this article. In this section, we reviewed studies employing TEM, cardiolipin, or CSA to elucidate the trends in mitochondrial content adaptation.

In **Table 3**, four studies (14, 24, 27, 28) were included, with two of them (14, 24) incorporating a healthy control group. Mitochondrial content (CSA) in the obese group did not significantly differ from that of the healthy control group. Although many studies have illustrated an association between lower muscle mitochondrial content and obesity (29–32), it is noteworthy that not all obese individuals exhibited reduced mitochondrial content (33–36). In addition to obesity, other factors such as age, physical activity and genetics may also influence mitochondrial content (17).

**Table 3:** Characteristics of skeletal muscle mitochondrial content following bariatric surgery.

Author (Year) (Reference)	Subjects information		Type of surgery & Time points	Site of skeletal muscle & Sample type	Main findings
	OG	LG			
S. Gancheva (2019) (24)	n=2 (TEM) (Gender & T2D status: N&A) n=45 (CSA) (F&M; T2D & Non-T2D)	n=13 (F&M)	RYGB, SG **PRE** **POST**: 2w, 12w, 24w, 52w	Vastus lateralis Ex-vivo tissue	**TEM (result from 2 subjects)** - OG[POST(2w, 52w) VS PRE]: ↓ - OG[POST(52w) VS POST(2w)]: ↑ **CSA** - OG[PRE] VS LG: → - OG[POST(2w) VS PRE]: ↓ - OG[POST(12w, 24w, 52w) VS PRE]: →
M. T. Lund (2018) (28)	n=14~16 (F&M; T2D) n=11~13 (F&M; Non-T2D)	N/A	RYGB **PRE**: ~2.5m, 1~2w **POST**: ~4.5m, ~19m	Vastus lateralis Ex-vivo tissue	**CSA** - OG[POST(~4.5m) VS POST(~19m) VS PRE (~2.5m) VS PRE(1~2w)]: →
M. D. Kristensen (2018) (14)	n=8 (F&M; Non-T2D) n=7 (F&M; T2D)	n=5 (M)	RYGB **PRE**: - A (the time of enrollment) - B (the time of ~8% of diet-induced weight loss) **POST**: 4m, 1.5y	Vastus lateralis Ex-vivo tissue	**CSA** - OG[(PRE(A, B) & POST(4m, 1.5y)] VS LG: → - OG[POST(1.5y) VS POST(4m) VS PRE(A) VS PRE(B)]: →
C.S. Mehnert (2017) (27)	n=6 (F&M; T2D status: N/A)	N/A	BPD **PRE** **POST**: 1y	Vastus lateralis Ex-vivo tissue	**Cardiolipidin** - OG(POST VS PRE): ↑

OG, Obese Group; LG, Lean Group; F, Female; M, Male; T2D, Type 2 Diabetes; Non-T2D, without Type 2 Diabetes; N/A, not applicable; RYGB, Roux-en-Y gastric bypass; BPD, biliopancreatic diversion; SG, sleeve gastrectomy; PRE, Pre-surgery; POST, Post-surgery; w, week or weeks; y, year or years; m, month or months; TEM, transmission electron microscopy; CSA, citrate synthase activity; ↑, upregulation; ↓, downregulation; →, no difference.

Regarding the effects of bariatric surgery on mitochondrial content, studies employing different methods have yielded varied results. Gancheva (24), using TEM, reported a decrease in mitochondrial content two weeks post-surgery, followed by a partial recovery that did not return to presurgical levels 52 weeks after surgery (RYGB or SG). This study also found an initial decrease in CSA at week 2, which then stabilized close to baseline levels from week 12 onward. Lund (28) and Kristensen (14), who did not assess CSA in the immediate post-surgery period as Gancheva, reported stable CSA levels after RYGB over time. In contrast, Mehnert’s study (27) indicated an increase in cardiolipin, reflecting mitochondrial content, one year after BPD procedure. These discrepancies across studies are likely due to differences in sample size and surgical procedures. Although Gancheva (24) used TEM, the gold standard measurement, the interpretation should be approached with caution given the small sample size of only two subjects. On the other hand, the CSA studies included larger cohorts and consistently showed stable trends across different investigations, making the results more compelling. For the cardiolipidin study, in addition to the relatively small sample size, other researchers suggested that BPD may exert greater metabolic improvements than RYGB (8), which may explain the more pronounced improvements of mitochondrial content following the surgery.

### 3.3 Mitochondrial dynamics remodeling

Mitochondria exist as interconnected and mobile networks, responding to cellular demands and environmental factors through ongoing fusion-fission dynamics (37). The fusion process is regulated by Mitofusin 1 (Mfn1), Mitofusin 2 (Mfn2), and Optic Atrophy 1 (OPA1) (38), while the fission process is mediated by dynamin-related protein 1 (Drp1) and Fission 1 (Fis1) (39). In addition to these biomarkers, mitochondrial dynamics can be directly assessed through morphological image processing. Abnormal mitochondrial dynamics are strongly associated with various diseases and pathologies (40). In the context of obesity, emerging evidence from animal studies indicates that obesity adversely affects muscle mitochondrial dynamics, characterized by increased expression of fission processes (41) and decreased expression of fusion processes (42), resulting in fragmented mitochondrial networks. In this section, we first reevaluated the differences in mitochondrial fission-fusion dynamics between obese and lean subjects in studies that included a healthy control group. Subsequently, we reviewed the effects of bariatric surgery on mitochondrial dynamics.

Seven studies (14, 24, 43–47) were included (**Table 4**), three of which incorporated a healthy control group (14, 24, 46). The majority of these studies corroborated the detrimental impact of obesity on muscle mitochondrial dynamics. Morphological assessments revealed more isolated mitochondria and fragmented mitochondrial networks in obese individuals (14, 46). Among the studies using biomarkers, Gancheva (24) and Kristensen (14) both demonstrated impaired mitochondrial dynamics in obese participants; however, their findings differed: Gancheva (24) reported reduced mitochondrial fusion (decreased Mfn2 and Opa1) with unchanged fission activity (unchanged pDrp1 and Fis1), while Kristensen (14) obeserved increased fission (elevated Drp1) alongside unchanged (unchanged Mfns and Opa1) or elevated fusion (elevated Mfns) in obese patients. Conversely, Kugler (46) found no difference in mitochondrial dynamics at the protein and mRNA levels of markers between obese and lean groups, despite the impairments in mitochondrial networks through imaging. Noably, Kugler’s study cultured myotubes derived from human skeletal muscle for 7 days before testing; however, the culture medium lacked appropriate components to simulate the extramyocellular lipid environment typical of *in vivo* skeletal muscle cells. Other *in vitro* studies have shown that excess palmitate was a primary factor inducing mitochondrial fragmentation in differentiated C2C12 muscle cells (41). Therefore, the absence of the extramyocellular lipid environment in Kugler’s medium may account for the discrepancies at the biomarker level.

**Table 4:** Characteristics of skeletal muscle mitochondrial dynamics following bariatric surgery.

Author (Year) (Reference)	Subjects information		Type of surgery & Time points	Site of skeletal muscle & Sample type	Main findings
	OG	LG			
B. A. Kugler (2020) (46)	n=6~7 (F; Non-T2D)	n=6 (F)	RYGB **PRE** **POST**: 1m, 7m	Vastus lateralis Myotubes (primarily cultured skeletal muscle satellite cell)	**Morphology** **Individual non-networked mitochondria** - OG[PRE, POST(1m)] VS LG: ↑ - OG[POST(7m)] VS LG: → **Number of branches per network** - OG[PRE, POST(1m, 7m)] VS LG: ↓ - OG[(POST(1m), POST(7m)) VS PRE]: → **Branch length per network** - OG[PRE, POST(1m, 7m)] VS LG: → - OG[POST(7m) VS PRE)]: ↑
					**Protein** **Mfn1, Mfn2, Opa1 short/long isoform, Drp1, Fis1** - OG[PRE, POST(1m, 7m)] VS LG: → - OG[POST(1m, 7m) VS PRE]: → **pDrps1** - OG[PRE, POST(1m, 7m)] VS LG: → - OG[POST(1m) VS PRE]: → - OG[POST(7m) VS PRE]: ↓
					**mRNA** **Mfn1, Mfn2, Opa1, Fis1** - OG[PRE, POST(1m, 7m)] VS LG: → - OG[POST(1m, 7m) VS PRE]: → **DNM1L (Drp1 gene)** - OG[PRE, POST(1m, 7m)] VS LG: → - OG[POST(1m) VS PRE]: → - OG[POST(7m) VS PRE]: ↓
S. Gancheva (2019) (24)	n=15~45 (F&M; T2D& Non-T2D)	n=10~13 (F&M)	RYGB, SG **PRE** **POST**: 2w, 12w, 24w, 52w	Vastus lateralis Ex-vivo tissue	**Protein** **Mfn2, Opa1** - OG(PRE) VS LG: ↓ - OG[(POST(2w, 12w, 24w) VS PRE]: → - OG[POST(52w) VS PRE]: ↑ **pDrp1** - OG(PRE) VS LG: → - OG[POST(2w, 12w, 24w, 52w) VS PRE]: → **Fis1** - OG(PRE) VS LG: → - OG[(POST(2w, 12w, 24w) VS PRE]: → - OG[POST(52w) VS PRE]: ↑
M. D. Kristensen (2018) (14)	n=8 (F&M; Non-T2D) n=7 (F&M; T2D)	n=5 (M)	RYGB **PRE**: - A (the time of enrollment) - B (the time of ~8% of diet-induced weight loss); **POST**: 4m, 1.5y	Vastus lateralis Ex-vivo tissue	**Morphology** **Percentage of fragmented and disorganized mitochondrial networks** - OG[T2D&Non-T2D(PRE(A))] VS LG: ↑ **(No quantitative results about mitochondrial remodeling after bariatic surgery)**
					**Protein** **Mfn1, Mfn2** - OG[T2D(PRE(A))] VS LG: → - OG[Non-T2D(PRE(A))] VS LG: ↑ - OG[T2D&Non-T2D(PRE(A) VS PRE(B) VS POST(4m) VS POST(1.5y))]: → **Opa1** - OG[T2D&Non-T2D(PRE(A)) VS LG: → - OG[T2D&Non-T2D(PRE(A) VS PRE(B) VS POST(4m) VS POST(1.5y))]: → **Fis1** - OG[(PRE(A))] VS LG: → - OG[(PRE(A) VS PRE(B) VS POST(4m))]: → - OG[(POST(1.5y) VS PRE(B))]: ↓ **Drp1** - OG[T2D&Non-T2D(PRE(A))] VS LG: ↑ - OG[T2D&Non-T2D(PRE(A) VS PRE(B) VS POST(4m) VS POST(1.5y))]: →
M. I. Hernández-Alvarez (2009) (45)	n=11 (F&M; Non-T2D) n=10 (F&M; T2D)	N/A	BPD **PRE** **POST**: 24m	Rectus abdominis Ex-vivo tissue	**mRNA** **Mfn2** - OG[Non-T2D(POST VS PRE)]: ↑ - OG[T2D(POST VS PRE)]: ↓
G. Gastaldi (2007) (47)	n=11~17 (F; T2D & Non-T2D)	N/A	RYGB **PRE** **POST**: 3m, 12m	Vastus lateralis Ex-vivo tissue	**mRNA** **Mfn2** - OG[POST(3m,12m) VS PRE]: ↑
G. Mingrone (2005) (43)	n=6 (F&M; Non-T2D)	N/A	BPD **PRE** **POST**: 2y	Vastus lateralis Ex-vivo tissue	**mRNA** **Mfn2** - OG(POST VS PRE): ↑
D. Bach (2005) (44)	n=6 (F&M; Non-T2D)	N/A	BPD **PRE** **POST**: 2y	Quadricep muscle Ex-vivo tissue	**mRNA** **Mfn2** - OG(POST VS PRE): ↑

OG, Obese Group; LG, Lean Group; F, Female; M, Male; T2D, Type 2 Diabetes; Non-T2D, without Type 2 Diabetes; N/A, not applicable; RYGB, Roux-en-Y gastric bypass; BPD, biliopancreatic diversion; SG, sleeve gastrectomy; PRE, Pre-surgery; POST, Post-surgery; w, week or weeks; y, year or years; m, month or months; Mfn1/2, Mitofusin1/2; OPA1, Optic Atrophy 1; Drp1, Dynaminrelated protein 1; Fis1, Fission; ↑, upregulation; ↓, downregulation; →, no difference.

As presented in **Table 4**, most studies suggested that bariatric surgery improved mitochondrial dynamics in human skeletal muscle. Mitochondrial morphology in myotubes from obese individuals showed improvement in “individual non-networked mitochondria” and “branch length per network” 7 months post-RYGB (46). Concurrently, a reduction in Drp1 phosphorylation and Drp1 gene (DNM1L) expression, indicative of decreased mitochondrial fission processes, was observed at the same time point (46). Kristensen (14) observed a diminished expression of the mitochondrial fission marker Fis1 at 1.5 years post-surgery, while other research (24, 44, 45, 47) reported a significant upregulation of mitochondrial fusion markers, particularly Mfn2 at protein or mRNA levels, from three months to two years after surgery. While the evidence above consistently confirmed the beneficial effects of bariatric surgery on mitochondrial dynamics, certain details should not be overlooked. In contrast to Kristensen’s (14) observation regarding Fis1, Gancheva (24) found an increase in Fis1 levels two years post-surgery. This discrepancy may arise from the different units of measurement used: Gancheva (24) reported the raw test results, while Kristensen (14) normalized results to CSA relative to the first time point (PRE(A)). Additionally, Hernández-Alvarez (45) investigated the effects of bariatric surgery on Mfn2 specifically in T2D obese patients, while other studies either included only Non-T2D obese patients or analyzed T2D and Non-T2D obese patients together. Interestingly, Hernández-Alvarez’s study (45) found that, unlike the increase in Mfn2 observed in Non-T2D obese patients postoperatively (43–45, 47), Mfn2 decreased in T2D obese patients after surgery. This suggests that the diabetic state may independently affect the outcomes of bariatric surgery, highlighting the need for further research to elucidate the underlying mechanisms.

### 3.4 Mitochondrial respiration remodeling

Mitochondrial respiration is the final stage of cellular respiration, during which substrates are oxidized to produce ATP in the coupled state or dissipated as heat in the uncoupled state. These processes are closed linked to the oxidative phosphorylation system (OXPHOS), the tricarboxylic acid (TCA) cycle, and uncoupling activities within the mitochondria. While the expression levels of specific proteins and genes may indicate mitochondrial respiratory function, direct assessment of oxygen consumption rates through respirometry provides a more comprehensive evaluation of mitochondrial respiration capacity (48). This section provided an overview of research on mitochondrial respiration remodeling following bariatric surgery with two parts: (i) the main components of mitochondrial respiration, and (ii) mitochondrial respiration capacity.

#### 3.4.1 Main components in mitochondrial respiration

The OXPHOS is responsible for converting energy from a series of redox reactions that transfer electrons from electron donors to electron acceptors into ATP, the primary energy form utilized by cells to preform their functions. Major components of OXPHOS include proteins of the electron transport chain (ETC) and ATP synthase. Seven studies (21, 23, 24, 43–46) in **Table 5** examined the expression of OXPHOS-related proteins and genes, with three of these studies also incorporating data from healthy individuals for comparison. Compared with the healthy control group, Gancheva (24) and Campbell (23) reported reduced levels of ETC protein in the obese group, a finding also supported by Antoun’s study (49), indicating decreased OXPHOS content in obesity. However, Kugler (46) did not find significant differences in ETC protein and ATPase expression, which may be attributed to the use of myotubes cultured *in vitro*. Regarding post-surgery effects, Gancheva (24) reported significant increases in Complex I (CI), Complex II (CII), Complex III (CIII), and Complex V (CV), while Complex IV (CIV) levels remained unchanged from pre-surgery, across different time points within 52 weeks. Findings related to CIV were consistent with research by Hernández-Alvarez (45), Mingrone (43) and Bach (44), who found no alterations in COX3, a protein involved in CIV assembly, at the mRNA level following bariatric surgery. In contrast to Gancheva’s findings of increased CI, Barberio (21) reported unchanged mRNA levels of CI subunits (NDUFB7 and NDUFA1) after surgery in T2D patients; however, the limited sample size (n=3) diminishes the credibility of this study. Kugler (46) also reported on multiple proteins from CI to CV but found that bariatric surgery did not affect any of these proteins. Once again, this outcome is likely attributed to the use of myotubes rather than ex vivo tissue. Only one study (23) reported that cytochrome c levels remained unaffected by bariatric surgery, although this study had a relatively short observation period of only three months post-surgery. In conclusion, bariatric surgery may enhance the expression of oxidative phosphorylation proteins in the skeletal muscle mitochondria of obese patients at various postoperative time points; however, the effects on specific proteins appear heterogeneous, necessitating further investigation.

**Table 5:** Characteristics of skeletal muscle oxidative phosphorylation system following bariatric surgery.

Author (Year) (Reference)	Sample size		Type of surgery & Timepoints	Site of skeletal muscle & Sample type	Main Finding
	OG	LG			
M. D. Barberio (2021) (21)	n=7 (F; Non-T2D) n=3 (F; T2D)	N/A	RYGB **PRE** **POST:** 1y	Vastus lateralis Ex-vivo tissue	**mRNA** **NDUFB7, NDUFA1** - OG[T2D(POST VS PRE)]: →
B. A. Kugler (2020) (46)	n=6~7 (F; Non-T2D)	n=6 (F)	RYGB **PRE** **POST**: 1m, 7m	Vastus lateralis Myotubes (primarily cultured skeletal muscle satellite cell)	**Protein** **Complex I-V (CI-V)** - OG[PRE, POST(1m), POST(7m)] VS LG: → - OG[(POST(1m), POST(7m)) VS PRE]: →
S. Gancheva (2019) (24)	n=15~45 (F&M; T2D& Non-T2D)	n=10~13 (F&M)	RYGB, SG **PRE** **POST**: 2w, 12w, 24w, 52w	Vastus lateralis Ex-vivo tissue	**Protein** **Complex I** - OG(PRE) VS LG: → - OG[POST(2w) VS PRE]: → - OG[POST(12w, 24w, 52w) VS PRE]: ↑ **Complex II** - OG(PRE) VS LG:↓ - OG[(POST(2w, 12w, 24w) VS PRE]: → - OG[POST(52w) VS PRE]: ↑ **Complex III** - OG(PRE) VS LG: ↓ - OG[POST(2w) VS PRE]: → - OG[POST(12w, 24w, 52w) VS PRE]: ↑ **Complex IV** - OG(PRE) VS LG: → - OG[POST(2w, 12w, 24w, 52w) VS PRE]: → **Complex V** - OG(PRE) VS LG: → - OG[POST(2w, 12w, 24w, 52w) VS PRE]: ↑
L. E. Campbell (2016) (23)	n=7 (F; T2D & Non-T2D)	n=4 (F)	RYGB **PRE** **POST**: 3m	Vastus lateralis Ex-vivo tissue	**Protein** **Cytochrome c** - OG(PRE, POST) VS LG: ↓ - OG(POST VS PRE)]: →
M. I. Hernández-Alvarez (2009) (45)	n=11 (F&M; Non-T2D) n=10 (F&M; T2D)	N/A	BPD **PRE** **POST**: 24m	Rectus abdominis Ex-vivo tissue	**mRNA** **COX3** - OG(POST VS PRE): →
G. Mingrone (2005) (43)	n=6 F&M; (Non-T2D)	N/A	BPD **PRE** **POST**: 2y	Vastus lateralis Ex-vivo tissue	**mRNA** **COX3** - OG(POST VS PRE): →
D. Bach (2005) (44)	n=6 (F&M; Non-T2D)	N/A	BPD **PRE** **POST**: 2y	Quadricep muscle Ex-vivo tissue	**mRNA** **COX3** - OG(POST VS PRE): →

OG, Obese Group; LG, Lean Group; F, Female; M, Male; T2D, Type 2 Diabetes; Non-T2D, without Type 2 Diabetes; N/A, not applicable; RYGB, Roux-en-Y gastric bypass; BPD, biliopancreatic diversion; SG, sleeve gastrectomy; PRE, Pre-surgery; POST, Post-surgery; w, week or weeks; y, year or years; m, month or months; NDUFB7/NDUFA1, genes encoding Complex I subunits; COX3, gene encoding Complex IV subunit; ↑, upregulation; ↓, downregulation; →, no difference.

Citrate synthase (CS) serves as the first rate-limiting enzyme in the TCA cycle (50). The preceding discussion has summarized CSA as a marker of mitochondrial content, indicating no disparity between obese and healthy lean individuals, with bariatric surgery showing no influence on CSA levels 12 weeks post-surgery (**Table 6**). This section reviewed the effects of obesity and bariatric surgery on CS content, revealing a trend similar to CSA. Despite differences in sample type, research by Kugler (46) and Campbell (23) collectively indicated comparable CS levels between healthy and obese individuals. Additionally, apart from the findings by Hernández-Alvarez et al. (45), which indicated an increase in skeletal muscle CS mRNA postoperatively in Non-T2D obese patients (but not in T2D obese patients), all other studies showed no significant difference in CS content at the protein or mRNA level before and after bariatric surgery. The reason for this discrepancy seems unrelated to the presence of T2D, as Mingrone (43) and Bach (44) did not observe an increase in CS mRNA postoperatively in Non-T2D patients. It is more likely to be attributed to differences in the skeletal muscle biopsy sites, as Hernández-Alvarez et al. (45) utilized the rectus abdominis muscle, while other studies used samples from the vastus lateralis or quadriceps muscles. The rectus abdominis is more involved in maintaining body posture, while the vastus lateralis is more directly engaged in body movement.

**Table 6:** Characteristics of skeletal muscle citrate synthase content following bariatric surgery.

Author (Year) (Reference)	Sample size		Type of surgery & Time points	Site of skeletal muscle & Sample type	Main Finding
	OG	LG			
B. A. Kugler (2020) (46)	n=6~7 (F; Non-T2D)	n=6 (F)	RYGB **PRE** **POST**: 1m, 7m	Vastus lateralis Myotubes (primarily cultured skeletal muscle satellite cell)	**Protein** **CS** - OG[PRE, POST(1m), POST(7m)] VS LG: → - OG[(POST(1m), POST(7m)) VS PRE]: →
L. E. Campbell (2016) (23)	n=7 (F; T2D & Non-T2D)	n=4 (F)	RYGB **PRE** **POST**: 3m	Vastus lateralis Ex-vivo tissue	**Protein** **CS** - OG(PRE, POST) VS LG: → - OG(POST VS PRE): →
M. I. Hernández-Alvarez (2009) (45)	n=11 (F&M; Non-T2D) n=10 (F&M; T2D)	N/A	BPD **PRE** **POST**: 24m	Rectus abdominis Ex-vivo tissue	**mRNA** **CS** - OG[Non-T2D(POST VS PRE)]:↑ - OG[T2D(POST VS PRE)]: →
G. Mingrone (2005) (43)	n=6 (F&M; Non-T2D)	N/A	BPD **PRE** **POST**: 2y	Vastus lateralis Ex-vivo tissue	**mRNA** **CS:** - OG[POST VS PRE]: →
D. Bach (2005) (44)	n=6 (F&M; Non-T2D)	N/A	BPD **PRE** **POST**: 2y	Quadricep muscle Ex-vivo tissue	**mRNA** **CS:** - OG[POST VS PRE]: →

OG, Obese Group; LG, Lean Group; T2D, Type 2 Diabetes; Non-T2D, without Type 2 Diabetes; N/A, not applicable; RYGB, Roux-en-Y gastric bypass; BPD, biliopancreatic diversion; PRE, Pre-surgery; POST, Post-surgery; w, week or weeks; y, year or years; m, month or months; CS, Citrate Synthase; ↑, upregulation; →, no difference.

Uncoupling proteins (UCPs) are mitochondrial membrane proteins that facilitate the dissipation of the electrochemical potential difference across the inner mitochondrial membrane. This process enables the transport of protons back into the mitochondrial matrix, leading to the release of energy as heat rather than converting it into ATP (51). The UCP family includes three isoforms: UCP1, UCP2, and UCP3 (52). UCP1 is predominantly found in brown adipose tissue (53), while UCP2 and UCP3 are primarily expressed in skeletal muscle tissue, with UCP3 as the predominant isoform (54, 55). To date, only two studies (47, 56) (**Table 7**) have examined UCP2 and/or UCP3 gene expression in skeletal muscle following bariatric surgery, and both consistently reported a downregulation of UCP2 and/or UCP3 mRNA in muscle tissue. These findings may suggest an alteration in mitochondrial energy metabolism in skeletal muscle and a reduction in mitochondrial uncoupling activity. The expression of UCP3 is closely regulated by intramuscular triglyceride content (56). The significant reduction in intramuscular triglyceride levels after bariatric surgery may help explain the downregulation of UCP3. Additionally, UCP3 and ROS interact with each other in a dynamic balance. On one hand, UCP3 reduces the proton gradient across the mitochondrial membrane, leading to decreased ROS production (57). On the other hand, superoxide can activate UCP3 expression (58). Therefore, the observed reduction in UCP3 post-surgery may reflect an improvement in oxidative stress within skeletal muscle.

**Table 7:** Characteristics of skeletal muscle uncoupling proteins following bariatric surgery.

Author (Year) (Reference)	Sample size		Type of surgery & Time points	Site of skeletal muscle & Sample type	Main Finding
	OG	LG			
G. Gastaldi (2007) (47)	n=11~17 (F; T2D & Non-T2D)	N/A	RYGB **PRE** **POST**: 3m, 12m	Vastus lateralis Ex-vivo tissue	mRNA **UCP3** - OG[POST(3m) VS PRE]: → - OG[POST(12m) VS PRE]: ↓
G. Mingrone (2003) (56)	n=11 (F&M; Non-T2D)	N/A	BPD **PRE** **POST**: 24m	Vastus lateralis Ex-vivo tissue	**mRNA** **UCP2, UCP3** - OG(POST VS PRE): ↓

OG, Obese Group; LG, Lean Group; F, Female; M, Male; T2D, Type 2 Diabetes; Non-T2D, without Type 2 Diabetes; N/A, not applicable; RYGB, Roux-en-Y gastric bypass; BPD, biliopancreatic diversion; PRE, Pre-surgery; POST, Post-surgery; w, week or weeks; y, year or years; m, month or months; UCP2/3, Uncoupling protein 2/3; ↓, downregulation; →, no difference.

#### 3.4.2 Mitochondrial respiratory capacity

Mitochondrial respiratory capacity is indicated by the oxygen consumption rates in structurally intact mitochondria. Unlike static assessments of molecular components, such as gene and protein expression or metabolite levels, oxygen consumption measurements provide a dynamic and real-time evaluations of cellular metabolism and mitochondrial function (48, 59). By stepwise modulation of coupling and substrate control, mitochondrial respiratory capacity can be comprehensively examined across various respiratory states using different types of biological samples. In skeletal muscle research, three types of samples—intact myotubes, permeabilized muscle fibers, and isolated mitochondria—serve distinct purposes. Intact myotubes preserve cellular structure and maintain mitochondria within the cellular environment, closely replicating respiratory function within the integrated cell. Both isolated mitochondria and mitochondria in permeabilized cells can directly interact with medium components, allowing for assessment of mitochondrial respiratory capacities specific to CI-CIV through stepwise additions (59).

As shown in **Table 8**, six studies (24, 28, 46, 60–62) were included in this section, employing various sample types and respiratory states. Three of these studies (24, 46, 62) included healthy control groups, revealing a consistent reduction in mitochondrial respiratory capacity associated with obesity, despite the differences in samples and respiratory states. Specifically, Kugler (46) observed significantly lower basal and maximal respiration rates in obese individuals compared with healthy controls using intact myotubes. When utilizing permeabilized muscle fibers, Gancheva (24) found reduced maximal uncoupled respiration in obesity while Vijgen (62) reported decreased oxidative phosphorylation respiration coupled to CI and CII. This decline in mitochondrial respiratory capacity in obesity was also corroborated by other studies (13), indicating that obesity impaired mitochondrial respiration.

**Table 8:** Characteristics of skeletal muscle mitochondrial respiratory capacity following bariatric surgery.

Author (Year) (Reference)	Sample size		Type of surgery & Time points	Site of skeletal muscle & Sample type	Unit	Main Finding
	OG	LG				
B. A. Kugler (2020) (46)	n=6 (F; Non-T2D)	n=6 (F)	RYGB **PRE** **POST**: 1m, 7m	Vastus lateralis Intact myotubes (primarily cultured skeletal muscle satellite cell)	pmol/min/μg protein	**Basal respiration oxygen consumption rate** - OG(PRE) VS LG: ↓ - OG[POST(1m, 7m)] VS LG: →; - OG[POST(1m, 7m) VS PRE]: → **ATP-production & Non-Mitochondrial respiration oxygen consumption rate** - OG[PRE, POST(1m, 7m)] VS LG: →; - OG[POST(1m, 7m) VS PRE]: → **Maximal respiration oxygen consumption rate** - OG [PRE, POST(1m)] VS LG: ↓; - OG [POST(7m)] VS LG: →; - OG [POST(1m, 7m) VS PRE]: →
S. Gancheva (2019) (24)	n=43~45 (F&M; Non-T2D&T2D)	n=13~14 (F&M)	RYGB, SG **PRE** **POST**: 2w, 12w, 24w, 36w, 52w	Vastus lateralis Permeabilized muscle fibers	pmol/s/mg wet tissue	**Maximal uncoupled respiration** - OG (PRE) VS LG: ↓ - OG[POST(2w) VS PRE]:↓ - OG[POST(12w, 24w, 52w) VS PRE]:→
M. T. Lund (2018) (28)	n=14~16 (F&M; T2D) n=11~13 (F&M; Non-T2D)	N/A	RYGB **PRE**: 2m, 1~2w **POST:** 4m, 19m	Vastus lateralis Permeabilized muscle fibers	pmol/s/mg wet tissue pmol/s/mg wet tissue/CS activity	**State 3 lipid OXPHOS capacity &** **Complex I and complex II linked state 3 OXPHOS capacity &** **Electron transfer system capacity** - OG[POST(4m, 19m) VS PRE(1~2w, 2m)]: →
M. Fernström (2016) (60)	n=10 (F; Non-T2D)	N/A	RYGB **PRE** **POST**: 6m	Vastus lateralis Isolated mitochondria	nmol/min/U CS	**State 3 respiration** - OG(POST VS PRE): ↑ **State 4 respiration** - OG(POST VS PRE): →
G. H. Vijgen (2013) (62)	n=8 (F&M; Non-T2D)	n=10 (M)	LAGB **PRE** **POST**: 1y	N/A Permeabilized muscle fibers	pmol/s/mg wet tissue/mtDNA	**State 2 respiration & State 4 respiration &** **Maximal respiration &** **State 3 respiration upon complex I and complex II substrates** - OG(POST VS PRE): → **State 3 respiration upon a lipid substrate for complex I &** **State 3 respiration upon a lipid substrate for complex I and complex II &** **State 3 respiration upon complex I substrates** - OG(POST VS PRE): ↑
S. Nijhawan (2013) (61)	n=7~13 (F&M; Non-T2D&T2D)	N/A	RYGB, SG **PRE** **POST**: 12w	Vastus lateralis Permeabilized muscle fibers	pmol/s/10 mg wet tissue/mL	**Basal respiration rate &** **Maximal respiration rate** - OG(POST VS PRE): →

OG, Obese Group; LG, Lean Group; F, Female; M, Male; T2D, Type 2 Diabetes; Non-T2D, without Type 2 Diabetes; N/A, not applicable; RYGB, Roux-en-Y gastric bypass; SG, sleeve gastrectomy; LAGB, laparoscopic adjustable gastric banding; PRE, Pre-surgery; POST, Post-surgery; w, week or weeks; y, year or years; m, month or months; OXPHOS, oxidative phosphorylation system; ↑, upregulation; ↓, downregulation; →, no difference.

However, the impact of bariatric surgery on mitochondrial respiratory capacity was inconsistent among different studies. Only Vijgen (62) and Fernström (60) reported enhancements in oxidative phosphorylation capacity (State 3 respiration) post-surgery, while other studies (24, 28, 46, 61) did not detect any improvement in skeletal muscle mitochondrial respiratory capacity. Notably, inconsistencies in experimental protocols, normalization methods, and terminology across studies have been observed. As depicted in **Table 8**, there were discrepancies in the units used to describe oxygen consumption rates, and especially, the substrates added to the medium across various respiratory states. Gnaiger and his colleagues have highlighted the lack of methodology harmonization in this area, advocating for the standardization of terminology related to mitochondrial respiratory states and rates, as well as experimental protocols (63). In addition to methodological issues, the level of physical activity following bariatric surgery is a significant factor influencing mitochondrial respiratory capacity. Both Woodlief (64) and Coen (65) reported that post-surgery exercise significantly improved skeletal muscle mitochondrial respiratory capacity compared with non-intervention groups. Although the six studies reviewed in this section did not include specific exercise interventions post-bariatric surgery, some research suggested that physical activity levels might increase following the procedure (66). None of these six studies reported on the physical activity level post-surgery. Consequently, variations in physical activity level after surgery may also account for the differences observed in study results.

### 3.5 Remodeling of mitochondrial markers in glucolipid metabolism

Following bariatric surgery, glucose and lipid metabolism in skeletal muscle improve significantly, as evidenced by a marked reduction in intramuscular triglycerides (27, 56, 67) and a substantial increase in skeletal muscle insulin sensitivity (43, 47, 56, 67, 68). As the primary site for the oxidation of carbohydrates and lipids in skeletal muscle, mitochondrial glucolipid markers may elucidate the mechanisms by which bariatric surgery enhances skeletal muscle metabolic health. This section reviewed the changes in mitochondrial markers associated with glucolipid metabolism following bariatric surgery.

In **Table 9**, eight studies (22, 28, 47, 67–71) reported six key glucolipid metabolism markers within skeletal muscle mitochondria following bariatric surgery. Overall, although bariatric surgery has a well-established positive effect on skeletal muscle health, the trends observed in mitochondrial biomarkers across current studies are inconsistent. Acetyl-CoA carboxylase 2 (ACC2) is the most frequently reported marker. This mitochondrial enzyme is crucial for regulating fatty acid oxidation capacity (72) and is modulated by phosphorylation (73). In its non-phosphorylated form, ACC2 catalyzes the carboxylation of acetyl-CoA to malonyl-CoA, which can inhibit the activity of carnitine palmitoyltransferase 1 (CPT1) through allosteric inhibition. This inhibition prevents the transport of long-chain fatty acyl-CoAs into the mitochondria, thereby reducing fatty acid oxidation (74). Three studies (67, 68, 71) found a significant decrease in ACC2 mRNA expression following bariatric surgery, with one study also reporting a marked reduction in its product, malonyl-CoA (67). This suggests that the transport of long-chain fatty acyl-CoAs into skeletal muscle mitochondria is enhanced post-surgery, leading to an increased capacity for lipid oxidation. Hinkley et al. (69) observed a similar trend regarding ACC2 phosphorylation. Their study found that the ratio of phosphorylated ACC2 increased one month post-surgery and returned to pre-surgery levels by seven months post-surgery, indicating that skeletal muscle mitochondria maintain or enhance their ability to oxidize fat following bariatric surgery. However, studies investigating CPT1B, the target of malonyl-CoA allosteric inhibition, did not indicate enhanced lipid oxidation capacity of skeletal muscle mitochondria after surgery. Three studies (47, 68, 70) demonstrated that, within 3-18 months post-bariatric surgery, CPT1B expression either decreased or remain unchanged. Additional findings include the unchanged activity of hydroxylacyl-CoA dehydrogenase, a key enzyme involved in fatty acid β-oxidation (75), following bariatric surgery (28). Similarly, the mRNA levels of pyruvate dehydrogenase kinase 4 (PDK4), an indicator of enhanced fat utilization (76), either deecreased or remained stable post-surgery (22, 70). Besides its role in lipid metabolism, PDK4 also inhibits glucose utilization (76). The unchanged or down-regulated mRNA levels of PDK4 following bariatric surgery suggest glucose metabolism was not further suppressed and may have been improved. Furthermore, hexokinase 2 (HK2), a mitochondrial enzyme that catalyzes the phosphorylation of D-glucose to D-glucose 6-phosphate (77), has also been shown to remain unchanged after bariatric surgery (70). These findings highlight the complexity of mitochondrial glucolipid adaptations following bariatric surgery and underscore the need for further research to clarify the relationships between these metabolic markers.

**Table 9:** Characteristics of skeletal muscle mitochondrial markers in glucolipid metabolism following bariatric surgery.

Author (Year) (Reference)	Sample size		Type of surgery & Time points	Site of skeletal muscle & Sample type	Main Finding	
	OG	LG				
M. T. Lund (2018) (28)	n=14~16 (F&M; T2D) n=11~13 (F&M; Non-T2D)	N/A	RYGB **PRE**: ~2m, 1~2w **POST**: ~4m, ~19m	Vastus lateralis Ex-vivo tissue	**Hydroxyacyl-CoA dehydrogenase activity** - OG[POST(4m, 19m) VS PRE(1~2w, 2m)]: →	
J. M. Hinkley (2017) (69)	n=6-8 (F; Non-T2D)	N/A	RYGB **PRE** **POST**: 1m, 7m	Vastus lateralis Primarily cultured skeletal muscle satellite cell	**Ratio of Phosphorylated ACC2 to total** - OG[POST(1m) VS PRE]: ↑ - OG[POST(7m) VS PRE]: →	
E. B. M. Nascimento (2015) (70)	n=8 (F&M; Non-T2D)	N/A	RYGB **PRE** **POST**: 6m	Vastus lateralis Primarily cultured skeletal muscle satellite cell	**mRNA** **PDK4,CPT1B, HK2** - POST VS PRE: →	
R. Barres (2013) (22)	n=5 (F; Non-T2D)	n=6 (F)	RYGB **PRE** **POST**: 6m	Vastus lateralis Ex-vivo tissue	**mRNA** **PDK4** - OG[PRE] VS LG: ↑ - OG[POST] VS LG:→ - OG[POST VS PRE]: ↓	
G. Gastaldi (2007) (47)	n=11~17 (F; T2D & Non-T2D)	N/A	RYGB **PRE** **POST**: 3m, 12m	Vastus lateralis Ex-vivo tissue	**mRNA** **CPT1B** - OG[POST(3m, 12m) VS PRE]: →	
G. Mingrone (2005) (71)	n=9 (F; Non-T2D)	N/A	BPD **PRE** **POST:** 14m	Vastus lateralis Ex-vivo tissue	**mRNA** **ACC2** - OG[POST VS PRE]: ↓	**Metabolite** **Malonyl-CoA** - OG[POST VS PRE]: ↓
R. Fabris (2004) (68)	n=10 (Gender: N/A; Non-T2D)	N/A	BPD **PRE** **POST**: 18 ± 2m	Quadricep muscle Ex-vivo tissue	**mRNA** **CPT1B & ACC2** - OG[POST VS PRE]: ↓	
G. Rosa (2003) (67)	n=12 (F&M; Non-T2D)	N/A	BPD **PRE** **POST**: 3y	Vastus lateralis Ex-vivo tissue	**mRNA** **ACC2** - OG[POST VS PRE]: ↓	

OG, Obese Group; LG, Lean Group; F, Female; M, Male; T2D, Type 2 Diabetes; Non-T2D, without Type 2 Diabetes; N/A, not applicable; RYGB, Roux-en-Y gastric bypass; BPD, biliopancreatic diversion; PRE, Pre-surgery; POST, Post-surgery; w, week or weeks; y, year or years; m, month or months; ACC2, Acetyl-CoA carboxylase 2; PDK4, pyruvate dehydrogenase kinase 4; CPT1B, carnitine palmitoyltransferase 1B; HK2, hexokinase 2; ↑, upregulation; ↓, downregulation; →, no difference.

## 4 Discussion

### 4.1 Possible mechanisms of mitochondrial remodeling induced by bariatric surgery

By reviewing of the included studies, we have clarified the impact of bariatric surgery on mitochondrial remodeling in skeletal muscle. Some discussion on the potential mechanisms of mitochondrial remodeling has already been provided in the mitochondrial dynamics section, specifically regarding changes in Mfn2, Drp1, and Fis1 that affect mitochondrial fusion and fission. From a broader perspective, we will discuss other possible mechanisms influencing skeletal muscle mitochondrial remodeling following bariatric surgery.

Weight loss is the most significant outcome of bariatric surgery; however, improved skeletal muscle mitochondrial function is not necessarily associated with weight loss. Mitochondrial respiratory capacity in skeletal muscle may remain unchanged or improve post-surgery, whereas caloric restriction-induced weight loss can lead to a decline in mitochondrial respiratory capacity (78, 79). One possible explanation for this discrepancy lies in their differing impacts on the gut microbiome, which has been shown to closely correlate with mitochondrial content and function in skeletal muscle (80). Research by Lahiri et al. (81) indicated that, despite similar weight loss, there were significant differences in gut microbiome composition between caloric restriction and surgical treatment. Bariatric surgery improved the obesity-associated gut microbiota composition, shifting it toward a lean microbiome phenotype. This finding also highlights the unique advantages of bariatric surgery in metabolic disease management.

Epigenetic modifications may also play a role in how bariatric surgery influences mitochondrial remodeling in skeletal muscle. Barres et al. (22) found that bariatric surgery reduced promoter methylation of proliferator-activated receptor γ coactivator-1α (PGC1α) in skeletal muscle, resulting in an increase in PGC1α mRNA expression. PGC1α is primarily associated with mitochondrial biogenesis and oxidative capacity, and enhanced expression of PGC1α has been shown to improve mitochondrial oxidative capacity (82). Other studies (45, 47, 69) have similarly confirmed the elevated expression of PGC1α post-surgery. Therefore, the downregulation of methylation in the PGC1α promoter, leading to increased PGC1α expression in skeletal muscle, may represent one of the mechanisms through which bariatric surgery enhances mitochondrial function.

### 4.2 Clinical implications

In general, bariatric surgery can improve mitochondrial function in skeletal muscle among obese patients; however, the impact of bariatric surgery on mitochondrial content and respiratory capacity remains controversial. Exercise has been recognized as the most effective behavioral intervention for enhancing mitochondrial health (83). To optimize the improvement of skeletal muscle mitochondrial function and metabolic health, a regular exercise program should be integrated into post-surgical care as a complementary therapy.

We have noted a close relationship between certain mitochondrial biomarkers affected by bariatric surgery and health indicators. These molecules may serve as potential therapeutic targets for further research. Kugler et al. (46) found that the level of phosphorylated Drp1 was significantly negatively correlated with glucose oxidation in response to insulin stimulation. Subsequent animal studies (84) demonstrated that skeletal muscle-specific Drp1 gene partial knockout significantly improved insulin sensitivity in obese mice. Another biomarker related to mitochondrial dynamics, Mfn2, exhibited a significant positive correlation with glucose utilization across several studies (43–45). Correspondingly, animal models with partial gene knockout of Mfn2 in skeletal muscle showed impaired glucose tolerance and insulin resistance (85), while overexpression models displayed moderate muscle hypertrophy without pathological changes (86). These two molecules have been preliminarily validated as potential therapeutic targets. In contrast, while ACC2 gene expression also changes significantly after surgery, its impact on metabolic health is limited. Rosa et al. (67) found that the post-surgical changes in ACC2 gene expression were positively correlated with variations in fasting insulin levels and intramuscular triglycerides. However, skeletal muscle-specific ACC2 knockout animal models did not exhibit metabolic abnormalities, indicating that the absence of ACC2 in skeletal muscle has a limited impact on metabolism (87). Given the broad and profound effects of bariatric surgery, many mitochondrial biomarkers related to the surgery could be carefully explored as potential therapeutic targets for obesity and related metabolic disorders.

### 4.3 Considerations for future research

As discussed above, there are certain limitations within current research. First, methodological inconsistencies in measuring mitochondrial respiratory capacity are prevalent, specifically in the following areas (63) (i) inconsistent terminology for mitochondrial respiratory states and rates across studies; (ii) substantial differences in experimental conditions (e.g., temperature, pH, substrate concentration) and methods (e.g., sample preparation, measurement instruments); and (iii) challenges in data normalization and standardization. These methodological discrepancies can significantly obscure the findings. Future researchers could refer to the standardized protocols proposed by MitoEAGLE (63), a global network project in mitochondrial physiology research, to address these methodological issues. Second, physical activity, a crucial factor influencing mitochondrial function, is often overlooked. Without clear data on patients’ physical activity levels before and after bariatric surgery, assessing the surgery’s impact on skeletal muscle mitochondrial remodeling becomes challenging. Future studies should adhere to standardized reporting of physical activity data. Physical activity levels can be measured using questionnaires such as the International Physical Activity Questionnaire (IPAQ) or, more simply, with smartphone step-tracking functions.

In addition to these primary issues, the influence of different surgical procedures and gender difference on skeletal muscle remodeling post-bariatric surgery warrants further investigation. Our review indicates that various surgical techniques may differentially impact mitochondrial content in skeletal muscle post-surgery. Although different metrics were used, BPD appears to promote a greater increase in mitochondrial content post-surgery compared with RYGB and SG. However, as the two most widely used bariatric procedures, the effects of SG and RYGB on skeletal muscle mitochondria post-surgery are unknown and require further investigation. Additionally, gender influences skeletal muscular mitochondrial function, with females typically exhibiting higher intramuscular lipid exposure yet stronger antioxidant capacities (88). Existing studies are either entirely female-focused or combine both genders, making it difficult to evaluate gender-specific effects on skeletal muscle remodeling post-surgery. Investigating these issues will not only provide a basis for optimizing surgical approaches but also support personalized postoperative management strategies, helping patients achieve more sustained improvements in metabolic health.

## 5 Conclusion

In summary, bariatric surgery impacts skeletal muscle through pathways related to mitochondrial function and induces mitochondrial remodeling in skeletal muscle in various ways. Among these, the improvement in mitochondrial dynamics is the most prominent. The effect of bariatric surgery on mitochondrial content is ambiguous, as it may be influenced by different types of surgical procedures. Regarding mitochondrial respiration, bariatric surgery may enhance the expression of markers within the OXPHOS while reducing the expression of UCP2/3, but the CS seems unaffected by surgical intervention. Moreover, discrepancies in methodology and terminology, along with the lack of physical activity information across studies, make it challenging to draw definitive conclusions about the remodeling of mitochondrial respiratory capacity post-surgery. The adaptations in mitochondrial markers associated with glucolipid metabolism following bariatric surgery are complex, displaying inconsistent trends.

Future studies should aim to standardize methodologies, enlarge sample sizes and consider the influence of factors such as physical activity levels, surgical procedures and gender difference on mitochondria in skeletal muscles following bariatric surgery. Further understanding the underlying mechanisms may not only elucidate the role of mitochondrial remodeling in the therapeutic benefits of bariatric surgery but also inform the development of new therapeutic strategies for obesity and related metabolic disorders.
