# Supplementary material for: Effect of bariatric surgery on mitochondrial remodeling in human skeletal muscle: a narrative review
Source: Front Endocrinol (Lausanne). 2024 Nov 25;15:1488715. doi: 10.3389/fendo.2024.1488715 (PMC11625573; doi:10.3389/fendo.2024.1488715)
Supplement: Supplementary file 1 [file DataSheet1.pdf]

Supplementary Material

Table S1 Search strategies

Database	Literature Search
PubMed	(((bariatric[Title] OR obesity[Title] OR metabolic[Title] OR "weight loss"[Title] OR "weight-loss"[Title]) AND (surg*[Title] OR oper*[Title] OR procedure*[Title])) OR ("gastric bypass"[Title/Abstract] OR "stomach bypass"[Title/Abstract] OR RYGB[Title/Abstract] OR "sleeve* gastrectom*"[Title/Abstract] OR "gastric sleev*"[Title/Abstract] OR "gastric band*"[Title/Abstract] OR "gastroileal bypass"[Title/Abstract] OR gastrojejunostom*[Title/Abstract] OR gastroplast*[Title/Abstract] OR "jejunoileal bypass"[Title/Abstract] OR "jejuno-ileal bypass"[Title/Abstract] OR "jejunoileac bypass"[Title/Abstract] OR "ileojejunal bypass"[Title/Abstract] OR "intestinal bypass"[Title/Abstract] OR "intestine bypass"[Title/Abstract] OR "ileum bypass"[Title/Abstract] OR "ileal bypass"[Title/Abstract] OR biliopancreatic[Title/Abstract] OR "bilio pancreatic"[Title/Abstract] OR "duodenal switch"[Title/Abstract] OR "biliointestinal anastomosis"[Title/Abstract] OR "intestinal anastomosis"[Title/Abstract] OR "intestine anastomosis"[Title/Abstract] OR "roux-en-y"[Title/Abstract] OR "roux y"[Title/Abstract])) AND (musc*[Title/Abstract])
Web of Science	(((TI=(bariatric OR obesity OR metabolic OR "weight loss" OR "weight-loss")) AND TI=(surg* OR oper* OR procedure*)) OR TS=("gastric bypass" OR "stomach bypass" OR RYGB OR "sleeve* gastrectom*" OR "gastric sleev*" OR "gastric band*" OR "gastroileal bypass" OR gastrojejunostom* OR gastroplast* OR "jejunoileal bypass" OR "jejuno-ileal bypass" OR "jejunoileac bypass" OR "ileojejunal bypass" OR "intestinal bypass" OR "intestine bypass" OR "ileum bypass" OR "ileal bypass" OR biliopancreatic OR "bilio pancreatic" OR "duodenal switch" OR "biliointestinal anastomosis" OR "intestinal anastomosis" OR "intestine anastomosis" OR "roux-en-y" OR "roux y")) AND TS=(musc*)
EBSCO	((TI (bariatric OR obesity OR metabolic OR "weight loss" OR "weight-loss") AND TI (surg* OR oper* OR procedure*)) OR SU ("gastric bypass" OR "stomach bypass" OR RYGB OR "sleeve* gastrectom*" OR "gastric sleev*" OR "gastric band*" OR "gastroileal bypass" OR gastrojejunostom* OR gastroplast* OR "jejunoileal bypass" OR "jejuno-ileal bypass" OR "jejunoileac bypass" OR "ileojejunal bypass" OR "intestinal bypass" OR "intestine bypass" OR "ileum bypass" OR "ileal bypass" OR biliopancreatic OR "bilio pancreatic" OR "duodenal switch" OR "biliointestinal anastomosis" OR "intestinal anastomosis" OR "intestine anastomosis" OR "roux-en-y" OR "roux y")) AND SU musc*
